# Association of systemic inflammation response index with mortality risk in older patients with hip fracture: a 10-year retrospective cohort study

**DOI:** 10.3389/fmed.2024.1401443

**Published:** 2024-05-22

**Authors:** Zhi Fang, Bo Gao, Zhicong Wang, Xi Chen, Mozhen Liu

**Affiliations:** ^1^Department of Orthopedics, People’s Hospital of Deyang City, Deyang, China; ^2^Department of Orthopedics, The First Affiliated Hospital of Dalian Medical University, Dalian, China

**Keywords:** systemic inflammation response index, mortality, hip fracture, older adults, predictor

## Abstract

**Objective:**

With a rapidly aging global population, the assessment of mortality risk following hip fracture in older adults has received increasing attention. Recently, the system inflammation response index (SIRI) has been identified as a novel prognostic marker to reflect both systemic inflammation and immune status. However, it is not yet known whether SIRI is a potential predictor of subsequent death in hip fracture patients. Therefore, this study aimed to investigate the association between SIRI and mortality in older patients with hip fracture.

**Methods:**

A total of 1,206 older hip fracture patients undergoing surgery between January 2013 and December 2022 were consecutively derived from our longitudinal database. Patients were divided into three groups according to SIRI tertiles, calculated as neutrophil × monocyte / lymphocyte. Survival status was obtained from medical records or telephone interviews, and the study outcome was all-cause mortality after hip fracture at the longest follow-up. Multivariate Cox proportional hazard model and restricted cubic spline (RCS) regression model were used to evaluate the association between SIRI and mortality. Moreover, a series of sensitivity analyses were conducted to further validate the robustness of the association.

**Results:**

During a median follow-up of 43.85 months, 337 patients (27.94%) died. After full adjustment, each unit increase in SIRI was significantly associated with a 2.2% increase in overall mortality (95% confidence interval [CI]: 1.001–1.042, *p* = 0.029). Similarly, compared with the first tertile of SIRI, the second and third tertile showed a 1.335-fold (95% CI: 1.011–1.762, *p* = 0.042) and 1.447-fold (95% CI, 1.093–1.917, *p* = 0.010) higher risk of death. Sensitivity analyses confirmed the stability of the association. Moreover, RCS analysis revealed a positive non-linear relationship between SIRI and mortality (*P* for nonlinearity = 0.021).

**Conclusion:**

High SIRI level at admission was significantly and positively associated with an increased risk of death, suggesting that SIRI may be an independent predictor of mortality in older patients with hip fracture.

## 1 Introduction

With a rapidly aging global population, hip fracture has become a public health concern due to its high morbidity, disability and mortality (1–5). Globally, hip fracture incidence was estimated to be 14.2 million, and the associated years lived with disability was 2.9 million in 2019 (5). Recently, two network meta-analyses, each including over 100 randomized controlled trails, reported that the 1-year mortality rates of femoral neck and intertrochanteric fracture after surgery were as high as 23.5 and 20.2%, respectively (6, 7). Therefore, to improve the prognosis of these patients, it is of great clinical significance to identify predictors for recognizing patients at high risk of death and for optimizing treatment strategies (8–10).

Attributing to easy availability and low cost in clinical practice, several blood indicators have been found to be associated with increased mortality in patients with hip fracture, including C-reactive protein (CRP) (11), albumin (10, 12), lymphocyte (10), neutrophil and monocyte (13). Based on these laboratory indicators, some integrated markers of systemic inflammation and immunity were further applied to the assessment of hip fracture prognosis, such as neutrophil-to-lymphocyte ratio (NLR) (14), monocyte-to-lymphocyte ratio (MLR) (15), neutrophil-to-albumin ratio (NAR) (16), and CRP-to-albumin ratio (CAR) (17). In addition, our previous studies also showed positive associations between platelet-to-lymphocyte ratio (PLR), systemic immune-inflammation index (SII) and mortality in older patients with hip fracture (18, 19).

More recently, the system inflammation response index (SIRI) has been considered as a novel marker that comprehensively reflects the balance between host inflammatory and immune status (20). Emerging studies indicated that high SIRI level was closely associated with poor prognosis in many diseases, including cancer (e.g., liver cancer, prostate cancer) (20, 21), cardiovascular disease (e.g., coronary artery disease, myocardial infarction) (22–24), cerebrovascular disease (e.g., stroke, intracerebral hemorrhage) (25, 26), respiratory disease (e.g., asthma) (27), and obesity (28). The SIRI is calculated from peripheral neutrophil, monocyte and lymphocyte (20). As described above, these blood indicators were significant predictors of death after hip fracture (10, 13–15). Furthermore, we also found that high monocyte level was associated with an increased risk of deep venous thrombosis (DVT) in older patients with hip fracture, thereby leading to a 3.21-fold increase in the 30-day mortality (29, 30). From these findings, we hypothesized that high SIRI level would be an independent predictor of subsequent death following hip fracture.

However, to our knowledge, no studies have investigated this relationship in hip fracture patients with a high risk of death. Our research team have established a hip fracture database that is widely used for prognosis studies (18, 19, 30–32). Therefore, using this database over a 10-year period (2013–2022), we aimed to explore the potential association between SIRI and mortality in older patients with hip fracture.

## 2 Materials and methods

### 2.1 Study design and patients

**Figure 1:**
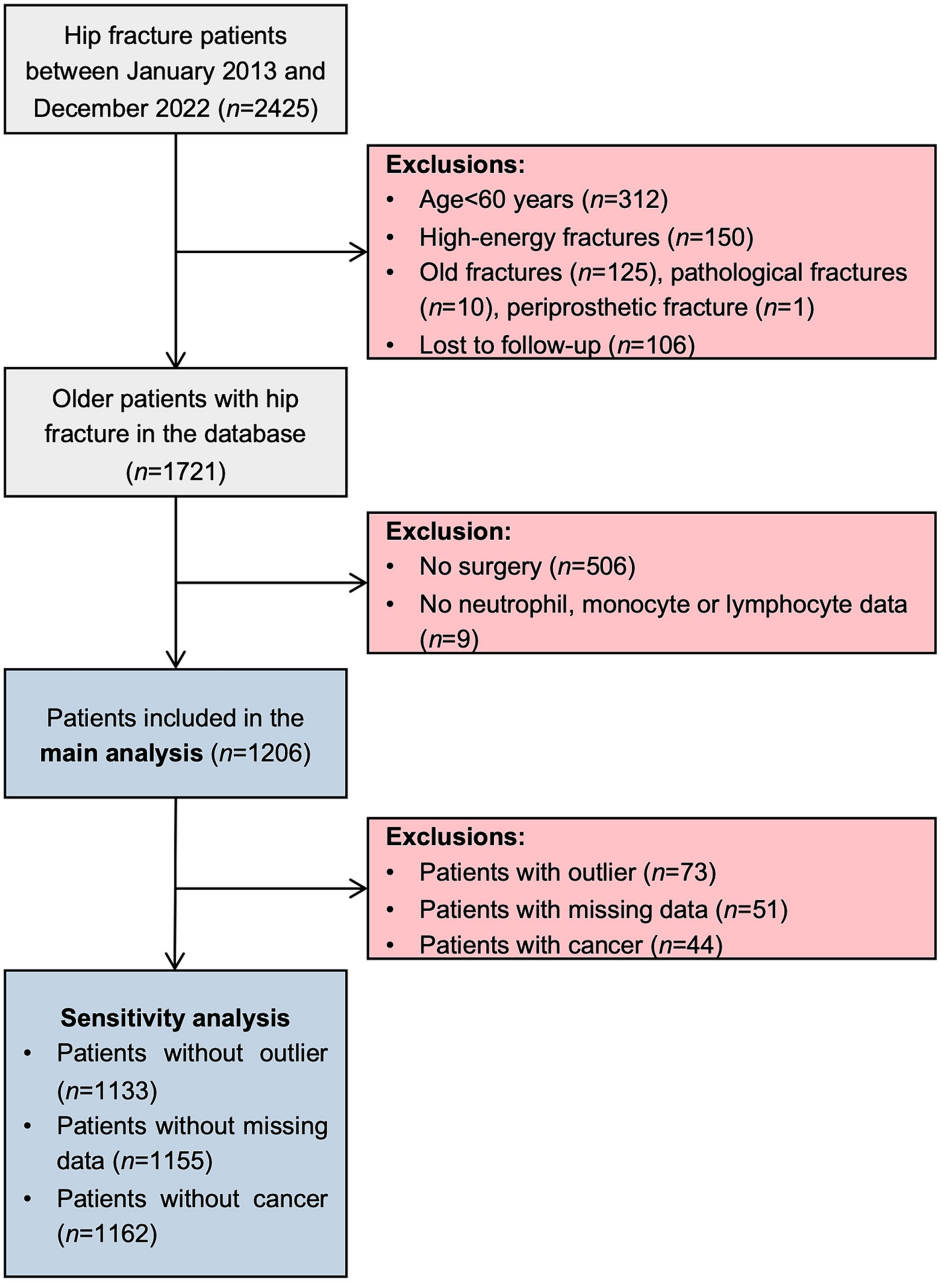
Patient screening flowchart.

This was a single-center, retrospective cohort study using our own hip fracture database, which is a longitudinal observational clinical database focusing on the prognosis of hip fracture (18, 19, 30–32). This electronic database belongs to Deyang Clinical Research Center for Orthopedics, Sports Medicine and Rehabilitation, which is affiliated with the orthopedic department of People’s Hospital of Deyang City that provides comprehensive medical care to approximately 3.5 million residents. This center serves as the main tertiary referral center for orthopedic patients in the district, thereby, hip fracture patients were admitted directly to the center or transferred from other hospitals for fracture treatment (e.g., surgery, conservative therapy), while other patients were admitted to the rehabilitation department for rehabilitation. After admission to the traditional orthopedic ward, all hip fracture patients were under evaluation for surgical treatment. If the patient’s physical condition permits, early surgical treatment is firstly recommended. Conservative treatment is proposed for hip fracture patients with a low survival probability. During hospitalization, a physical therapist started a rehabilitation program as early as possible to prevent complications from prolonged immobility. As for the inclusion criteria, all patients admitted to the orthopedic ward with a diagnosis of hip fracture were automatically entered into the database. Between 1 January 2013 and 31 December 2022, 2,425 patients were initially included. Subsequently, patients meeting any of the following criteria were excluded from the database: (1) age < 60 years; (2) fracture due to high-energy trauma (e.g., traffic accident, fall from higher than standing height); (3) old fracture (> 3 weeks after injury), pathological fracture, periprosthetic fracture; (4) lost to follow-up. In this study, patients who did not undergo surgery, and with missing data on neutrophil, monocyte or lymphocyte were further excluded. Considering 20 variables in multivariate regression model described below, the sample size in this study should be at least 716 (200 events) based on the rule of thumb of 10 events per variable (EPV) (33). As illustrated in Figure 1, our sample size was sufficient to yield valid results. The study protocol was approved by our Institutional Ethics Committee (Ethical approve number: 2022–04-040-K01). The study complied with the Declaration of Helsinki, and all patients gave written informed consent for the use of their clinical data for research purposes. This study was conducted and reported in line with Strengthening The Reporting Of Cohort Studies in Surgery (STROCSS) 2021 guideline (34).

### 2.2 Data collection and definition

The hip fracture database is linked to the Medical Data Center of our hospital, which is a research data repository that receives real-time data from hospital information system (HIS), electronic medical record (EMR) system, picture archiving and communication system (PACS), laboratory information system (LIS), and clinical information system (CIS). Last accessed on 31 December 2023, about 6.4 billion medical data and 24.5 million hospital visit records (including inpatient, outpatient and emergency) were deposited into the big data center. Data in the database were automatically populated from the Medical Data Center, and then checked for accuracy by a trained research assistant via review of medical records. The following data were extracted.

1. Demographics included age, sex, height, weight, marital status, smoking, year of admission and length of stay. Body mass index (BMI) was calculated as body weight/height^2^ (kg/m^2^), and classified on the basis of 2021 Chinese guideline: underweight (BMI < 18.5 kg/m^2^), normal weight (BMI: 18.5–23.9 kg/m^2^), overweight and obese (BMI ≥ 24.0 kg/m^2^) (35). Marital status was grouped as widowed and other (including single, married, divorced). Smoking was categorized as smoking (including former smoking) and never smoking. Due to the significant impact of the COVID-19 pandemic on the excess mortality, year of admission was divided into two periods: 2013–2019 and 2020–2022 (36). Length of stay was calculated based on admission and discharge dates.
2. Comorbidities included hypertension, diabetes mellitus, coronary heart disease, atrial fibrillation, heart failure, peripheral vascular disease, cerebrovascular disease, Alzheimer disease, chronic pulmonary disease, connective tissue disease, liver disease, hemiplegia, chronic kidney disease, and cancer. Information concerning comorbidities was obtained from admission diagnose and previous medical history.
3. Type of hip fracture included femoral neck fracture and intertrochanteric fracture. All fractures were radiographically confirmed by X-ray and/or computed tomography (CT) examination.
4. Surgical data included time to surgery, surgical procedure, and anesthetic type. Time to surgery was defined as the time (days) from hospital admission to the start of surgery. According to the surgical records, surgical procedure was categorized as internal fixation (cannulated screw, dynamic hip screw, and intramedullary nail fixation), hemiarthroplasty and total hip arthroplasty. Anesthetic type used for hip fracture surgery was classified as general anesthesia (intravenous, inhalation anesthesia), and regional anesthesia (spinal, epidural, and combined spinal-epidural anesthesia).
5. First laboratory data after admission included neutrophil, monocyte and lymphocyte. All blood samples were collected within 24 h of admission, and analyzed by the clinical laboratory of our hospital, which has been accredited by the China National Accreditation Service for Conformity Assessment (CNAS) in accordance with ISO 15189: 2012 (Registration No. CNAS MT0754). Briefly, blood cell counts were measured using automatic hematology analyzers (Sysmex XN2000, Kobe, Japan), and the reference ranges were 1.8–6.3 × 10^9^/L for neutrophil, 0.1–0.6 × 10^9^/L for monocyte, and 1.1–3.2 × 10^9^/L for lymphocyte.

### 2.3 Definition of SIRI

The SIRI was calculated using the following formula: SIRI = neutrophil × monocyte / lymphocyte, and its unit is expressed as 10^9^/L (27). Patients were divided into three groups according to SIRI tertiles, and the lowest tertile served as the reference group.

### 2.4 Flow-up and mortality

The outcome of interest was all-cause mortality after hip fracture at the longest follow-up duration. Death information was collected as follows. In-hospital deaths were automatically identified from the Medical Data Center described above, and verified with death certificates. Surviving patients were followed up by telephone interviews every year until death. The follow-up contents included survival status, date of death and cause of death, if applicable. The last telephone follow-up time was April, 2023. Beside this, survival information after discharge was also obtained from the most recent clinical records, including outpatient visits, emergency visits and readmission records. If survival status could not be determined by any of the above methods, patients were considered lost to follow-up, and the lost to follow-up rate for our database was 5.80% (106/1,827). In this study, survival time was defined as the time interval (months) from hospital admission to death (event) or last follow-up (censored), whichever occurred first.

### 2.5 Statistical analysis

Prior to analysis, all data were checked for missing values, and 4.23% of BMI data (*n* = 51) were missing. We then imputed missing values using low-rank matrix approximation method (37). Afterwards, continuous variables were presented as median (interquartile range [IQR]) according to the results of Shapiro–Wilk normality test, whereas categorical variables as numbers (percentages). Differences among the tertile groups were compared by Wilcoxon rank-sum test or Pearson’s chi-square test. The 95% confidence interval (CI) for mortality rate was calculated using the Wilson score method, and trend across tertiles was analyzed using the Cochran-Armitage trend test. Moreover, violin plots were used to compare the levels of neutrophil, monocyte, lymphocyte and SIRI between patients who were alive and dead.

For time-to-event analysis, Kaplan–Meier curve with log-rank test was plotted to visualize the survival differences, and median follow-up time was calculated using the reverse Kaplan–Meier method. Univariate Cox proportional hazards models were applied to identify factors associated with death, and factors with a *p* value < 0.1 in the univariate analyses were further included in the multivariate Cox regression (Supplementary Table S1). Hazard ratio (HR) and 95% *CI* were calculated to estimate the risk of death. To assess the association between SIRI and mortality, three models were constructed to adjust for potential confounding factors. Model 1 was adjusted for demographics (age, sex, BMI, marital status, smoking, year of admission, length of stay), model 2 was adjusted for demographics, and comorbidities (diabetes mellitus, coronary heart disease, atrial fibrillation, cerebrovascular disease, Alzheimer disease, chronic pulmonary disease, chronic kidney disease, cancer), model 3 was adjusted for demographics, comorbidities, type of hip fracture, and surgical data (time to surgery, surgical procedure, anesthetic type). The SIRI was included in separate models as both a continuous and a categorical variable. To test for linear trend, the median value of SIRI was assigned to each tertile (first tertile: 1.72, second tertile: 3.78, third tertile: 7.74), and this value was modeled as a continuous variable. The proportional hazard assumption was checked using Schoenfeld residuals, and no violations were detected. Multicollinearity was evaluated using variance inflation factor (VIF), and no significant multicollinearity was found between included variables for any model (Supplementary Table S2).

To validate the robustness of the main findings, multiple sensitivity analyses were performed. Firstly, we repeated the same analyses after removing outlier individuals, identified as those with SIRI values outside the 1.5 × IQR. Secondly, to reduce bias due to missing data, patients with missing data were excluded from the study. Thirdly, hip fracture patients with a history of any cancer are at increased risk of death (9), therefore, these patients were further excluded (Figure 1). Moreover, restricted cubic spline (RCS) regression model with three knots at the 10th, 50th, and 90th percentiles was conducted to further explore the dose–response relationship between SIRI and mortality.

All reported *p* values are two-sided, and *p* < 0.05 was considered statistically significant. Statistical analysis was performed using JMP Pro software (version 17.0.0; SAS Institute Inc., Cary, NC, United States), GraphPad Prism (version 9.1.1; GraphPad Software, San Diego, California, United States) and R statistical software (version 4.2.2; R Project for Statistical Computing).

## 3 Results

### 3.1 Patient characteristics

A total of 1,206 older patients with hip fracture were included in the main analysis, with a median age of 79.00 years and 33.25% were male. The first and second tertile values of SIRI were 2.56 and 5.22, and patients were then divided into three groups: first tertile (≤ 2.56, *n* = 402), second tertile (2.57–5.22, *n* = 402), and third tertile (> 5.22, *n* = 402). Patient characteristics stratified by tertiles of SIRI are listed in Table 1. Statistically significant differences were observed in age, sex, smoking, year of admission, cerebrovascular disease, chronic pulmonary disease, type of hip fracture, time to surgery, and surgical procedure (all *p* < 0.05).

**Table 1:** Patient characteristics stratified by tertiles of systemic inflammation response index.

Variables	Overall (*n* = 1,206)	First tertile (*n* = 402)	Second tertile (*n* = 402)	Third tertile (*n* = 402)	*p*
**Demographics**					
Age (years), median (IQR)	79.00 (72.00–84.00)	77.00 (69.00–82.25)	79.00 (72.00–85.00)	80.00 (74.00–85.00)	<0.001
Sex, male, *n* (%)	401 (33.25)	108 (26.87)	157 (39.05)	136 (33.83)	0.001
**Body mass index (Kg/m** ^**2**^ **), *n* (%)**					0.128
Underweight	206 (17.08)	54 (13.43)	72 (17.91)	80 (19.90)	
Normal weight	682 (56.55)	238 (59.20)	219 (54.48)	225 (55.97)	
Overweight and obese	318 (26.37)	110 (27.36)	111 (27.61)	97 (24.13)	
Marital status, widowed, *n* (%)	292 (24.21)	92 (22.89)	97 (24.13)	103 (25.62)	0.663
Smoking, *n* (%)	256 (21.23)	68 (16.92)	99 (24.63)	89 (22.14)	0.024
**Year of admission, *n* (%)**					<0.001
2013–2019	692 (57.38)	272 (67.66)	228 (56.72)	192 (47.76)	
2020–2022	514 (42.62)	130 (32.34)	174 (43.28)	210 (52.24)	
Length of stay (days), median (IQR)	14.00 (11.00–19.00)	13.00 (11.00–19.00)	14.00 (11.00–19.00)	14.00 (11.00–19.00)	0.220
**Comorbidities, *n* (%)**					
Hypertension	469 (38.89)	147 (36.57)	166 (41.29)	156 (38.81)	0.388
Diabetes mellitus	253 (20.98)	76 (18.91)	85 (21.14)	92 (22.89)	0.381
Coronary heart disease	77 (6.38)	25 (6.22)	30 (7.46)	22 (5.47)	0.507
Atrial fibrillation	27 (2.24)	7 (1.74)	9 (2.24)	11 (2.74)	0.635
Heart failure	25 (2.07)	7 (1.74)	11 (2.74)	7 (1.74)	0.520
Peripheral vascular disease	18 (1.49)	7 (1.74)	4 (1.00)	7 (1.74)	0.602
Cerebrovascular disease	134 (11.11)	32 (7.96)	51 (12.69)	51 (12.69)	0.048
Alzheimer disease	32 (2.65)	12 (2.99)	13 (3.23)	7 (1.74)	0.370
Chronic pulmonary disease	231 (19.15)	58 (14.43)	74 (18.41)	99 (24.63)	0.001
Connective tissue disease	23 (1.91)	7 (1.74)	10 (2.49)	6 (1.49)	0.562
Liver disease	20 (1.66)	6 (1.49)	3 (0.75)	11 (2.74)	0.083
Hemiplegia	14 (1.16)	5 (1.24)	7 (1.74)	2 (0.50)	0.253
Chronic kidney disease	51 (4.23)	16 (3.98)	21(5.22)	14 (3.48)	0.450
Cancer	44 (3.65)	15 (3.73)	9 (2.24)	20 (4.98)	0.117
**Type of hip fracture, *n* (%)**					<0.001
Femoral neck fracture	618 (51.24)	257 (63.93)	196 (48.76)	165 (41.04)	
Intertrochanteric fracture	588 (48.76)	145 (36.07)	206 (51.24)	237 (58.96)	
Time to surgery (days), median (IQR)	5.61 (3.83–7.68)	5.03 (3.69–7.05)	5.60 (3.80–7.53)	5.89 (3.98–8.10)	0.009
**Surgical procedure, *n* (%)**					<0.001
Internal fixation	665 (55.14)	193 (48.01)	224 (55.72)	248 (61.69)	
Hemiarthroplasty hip arthroplasty	336 (27.86)	116 (28.86)	111 (27.61)	109 (27.11)	
Total hip arthroplasty	205 (17.00)	93 (23.13)	67 (16.67)	45 (11.19)	
**Anesthetic type, *n* (%)**					0.178
General anesthesia	894 (74.13)	310 (77.11)	297 (73.88)	287 (71.39)	
Regional anesthesia	312 (25.87)	92 (22.89)	105 (26.12)	115 (28.61)	

IQR, interquartile range; n, number.

The *p* value refers to the comparison between the three groups.

### 3.2 All-cause mortality

**Figure 2:**
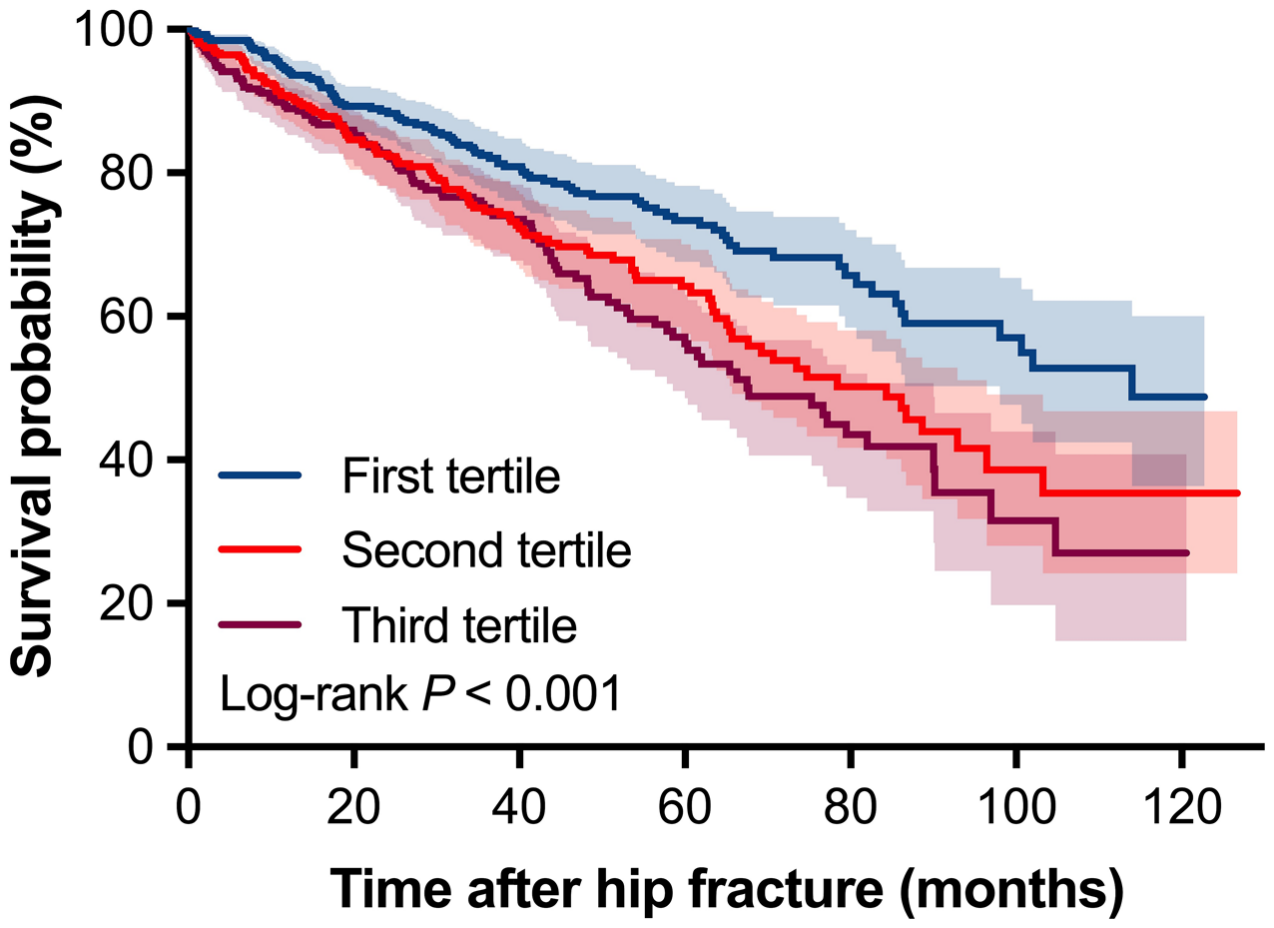
Kaplan–Meier survival curve for overall mortality stratified by tertiles of systemic inflammation response index.

**Figure 3:**
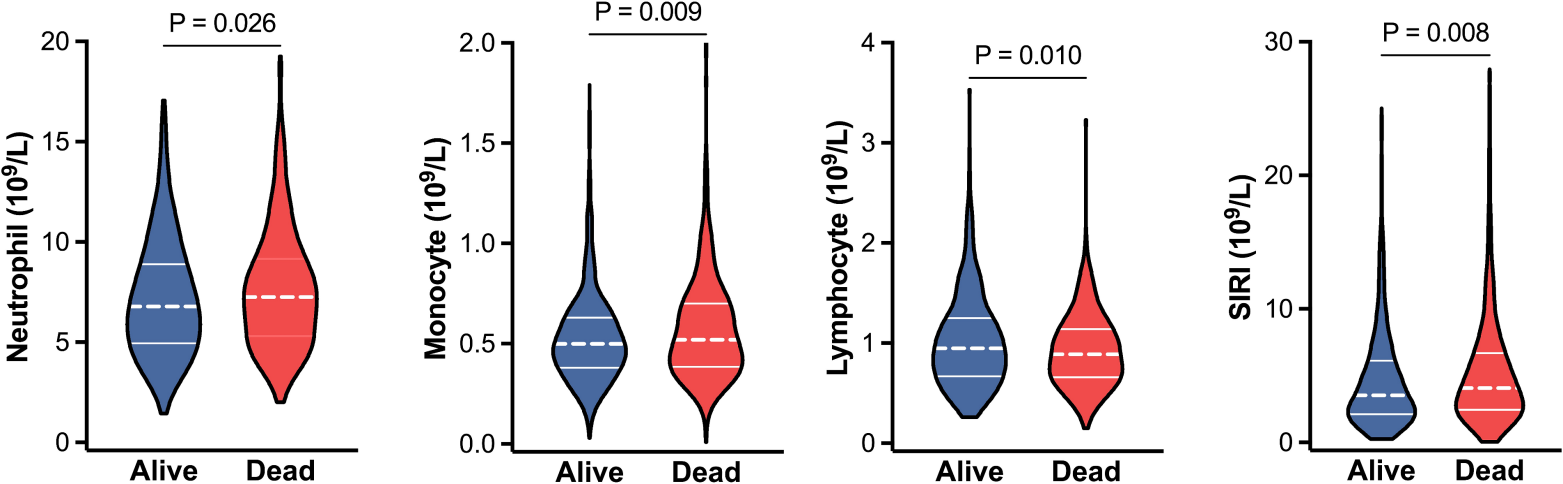
Violin plots for the levels of neutrophil, monocyte, lymphocyte and SIRI between alive (blue) and dead (red) patients. The dashed lines within each violin indicate medians, and solid lines indicate interquartile range. Abbreviations: SIRI, systemic inflammation response index.

During a median follow-up of 43.85 months (IQR: 39.73–46.71 months), 337 patients (27.94, 95% *CI*: 25.48–30.54%) died from any cause (Table 2). Evaluating groups by tertiles, a higher SIRI tertile exhibited higher mortality rates, and this increased mortality from first tertile to third tertile was statistically significant (*P* for trend = 0.003). Consistently, Kaplan–Meier survival curve also showed significant differences in survival probabilities among SIRI tertiles, and the lowest survival rate was observed in the third tertile (log-rank *χ*^2^ = 19.537, *p* < 0.001, Figure 2). In addition, patients who were dead had higher levels of neutrophil and monocyte, and lower level of lymphocyte than those who were alive (all *p* < 0.05, Figure 3). As expected, the SIRI levels were significantly higher in dead patients than in alive patients (4.08 [2.46–6.68] vs. 3.55 [2.12–6.12], *p* = 0.008, Figure 3).

**Table 2:** Association between systemic inflammation response index and overall mortality in the main analysis (*n* = 1,206).

	Continuous SIRI (per unit)	First tertile	Second tertile	Third tertile	*P* for trend
Death events, *n*	337	92	115	130	
Mortality rate (%, 95 CI)	27.94 (25.48–30.54)	22.89 (19.05–27.24)	28.61 (24.41–33.21)	32.34 (27.95–37.06)	0.003
Crude HR (95% CI)	1.032 (1.013–1.049), *p* = 0.002	1.0 (ref)	1.544 (1.181–2.019), *p* = 0.002	1.796 (1.372–2.351), *p* < 0.001	<0.001
Adjusted HR (95% CI) Model 1	1.027 (1.009–1.045), *p* = 0.003	1.0 (ref)	1.393 (1.060–1.831), *p* = 0.017	1.509 (1.145–1.987), *p* = 0.003	0.007
Model 2	1.026 (1.007–1.046), *p* = 0.007	1.0 (ref)	1.380 (1.049–1.815), *p* = 0.021	1.482 (1.124–1.955), *p* = 0.005	0.010
Model 3	1.022 (1.001–1.042), *p* = 0.029	1.0 (ref)	1.335 (1.011–1.762), *p* = 0.042	1.447 (1.093–1.917), *p =* 0.010	0.018

SIRI, systemic inflammation response index; n, number; CI, confidence interval; HR, hazard ratio; ref, reference.

Model 1: adjusted for demographics (age, sex, body mass index, marital status, smoking, year of admission, length of stay).

Model 2: adjusted for demographics, and comorbidities (diabetes mellitus, coronary heart disease, atrial fibrillation, cerebrovascular disease, Alzheimer disease, chronic pulmonary disease, chronic kidney disease, cancer).

Model 3: adjusted for demographics, comorbidities, type of hip fracture, and surgical data (time to surgery, surgical procedure, anesthetic type).

### 3.3 Association between SIRI and mortality

As shown in Table 2, Cox regression analysis showed that continuous SIRI was significantly associated with an increased risk of mortality in all multivariate models (*p* < 0.05 for model 1, model 2 and model 3). In the fully adjusted models (model 3), compared with the first tertile of SIRI, the second and third tertile had a 1.335-fold (95% CI: 1.011–1.762, *p* = 0.042) and 1.447-fold (95% CI: 1.093–1.917, *p* = 0.010) higher risk of death, and this trend reached statistical significance (*p* = 0.018). However, there were no obvious differences between second and third tertile. Even after removing patients with outlier, missing data or cancer, this association remained significant (all *p* < 0.05, Table 3).

**Table 3:** Association between systemic inflammation response index and overall mortality in the sensitivity analysis.

	Patients without outlier (*n* = 1,133)		Patients without missing data (*n* = 1,155)		Patients without cancer (*n* = 1,162)	
	HR (95% CI)	*p*	HR (95% CI)	*p*	HR (95% CI)	*p*
Crude model	1.087 (1.045–1.128)	<0.001	1.033 (1.013–1.050)	<0.001	1.037 (1.017–1.054)	<0.001
Model 1	1.046 (1.005–1.089)	0.029	1.023 (1.003–1.040)	0.014	1.025 (1.006–1.045)	0.012
Model 2	1.046 (1.004–1.089)	0.032	1.022 (1.001–1.041)	0.025	1.025 (1.005–1.046)	0.016
Model 3	1.045 (1.003–1.089)	0.037	1.021 (1.001–1.041)	0.044	1.024 (1.001–1.045)	0.041

HR, hazard ratio; CI, confidence interval.

Model 1: adjusted for demographics (age, sex, body mass index, marital status, smoking, year of admission, length of stay).

Model 2: adjusted for demographics, and comorbidities (diabetes mellitus, coronary heart disease, atrial fibrillation, cerebrovascular disease, Alzheimer disease, chronic pulmonary disease, chronic kidney disease, cancer).

Model 3: adjusted for demographics, comorbidities, type of hip fracture, and surgical data (time to surgery, surgical procedure, anesthetic type).

### 3.4 Dose-response relationship between SIRI and mortality

**Figure 4:**
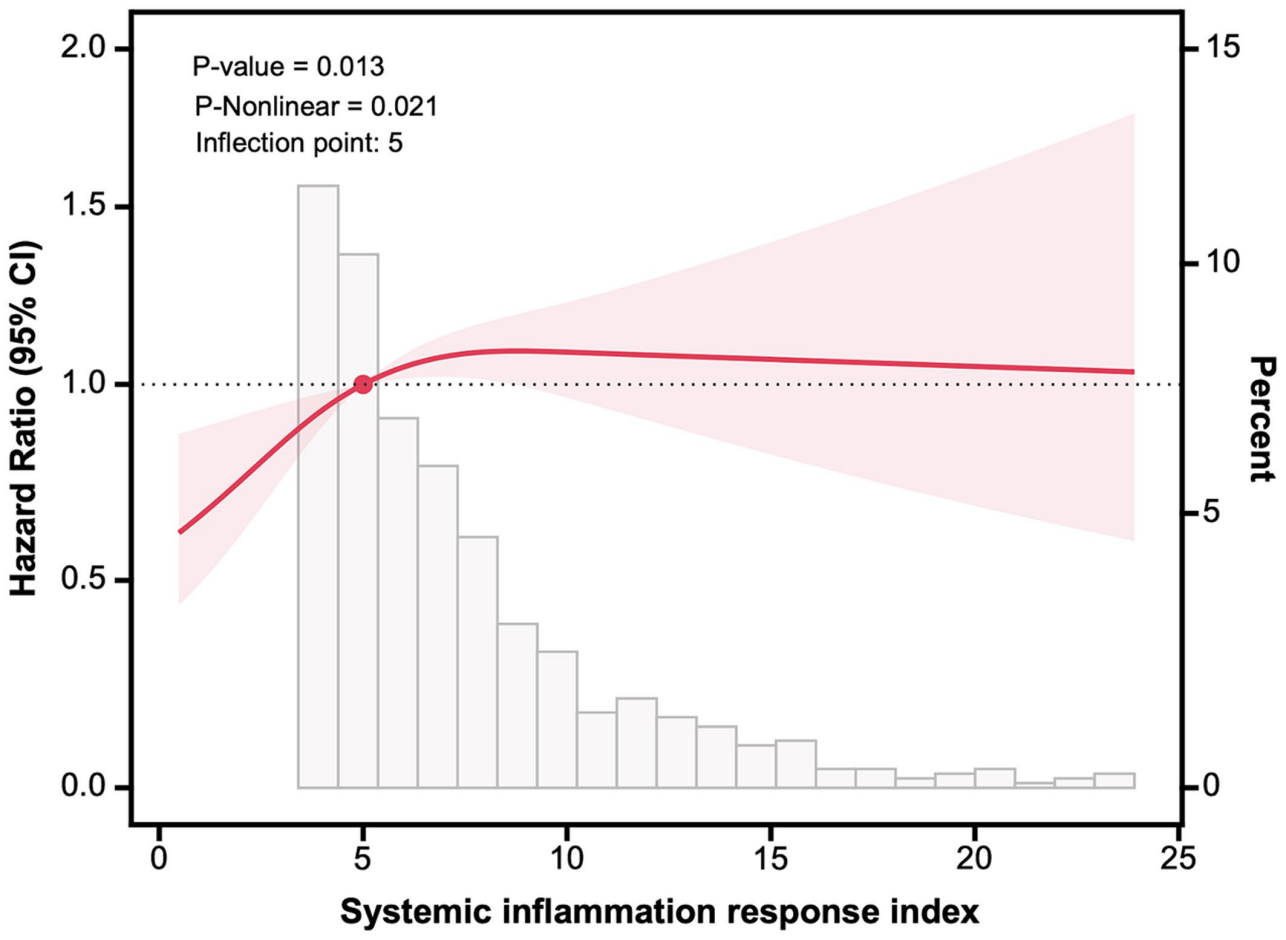
Restricted cubic spline analysis between systemic inflammation response index and overall mortality. Adjusted for demographics (age, sex, body mass index, marital status, smoking, year of admission, length of stay), comorbidities (diabetes mellitus, coronary heart disease, atrial fibrillation, cerebrovascular disease, Alzheimer disease, chronic pulmonary disease, chronic kidney disease, cancer), type of hip fracture, and surgical data (time to surgery, surgical procedure, anesthetic type).

As shown in Figure 4, RCS analysis revealed a positive non-linear relationship between SIRI and mortality (*P* for nonlinearity = 0.021), and the inflection point was 5.0. Below this point, each unit increase in SIRI corresponded to a 15.2% increase in death risk (95% CI: 1.029–1.290, *p* = 0.014). However, no significant difference was observed above this point (HR = 1.005, 95% CI: 0.972–1.033, *p* = 0.739).

## 4 Discussion

In this study, we assessed the relationship between SIRI and mortality in older patients with hip fracture, and found that a higher SIRI level at admission indicated a higher risk of mortality. Even after adjusting for patient demographics, comorbidities, type of hip fracture and surgical data, this positive association remained significant. For instance, patients in the second and third SIRI tertile showed a 1.335-fold and 1.447-fold higher risk of death compared with those in the first tertile, and this increasing trend from first tertile to third tertile was statistically significant. In agreement with our findings, elevated SIRI was positively associated with 90-day all-cause mortality (third vs. first tertile: HR = 1.41, 95% CI: 1.18–1.68, *P* for trend = 0.001), and 1-year all-cause mortality in older patients with heart failure (HR = 1.19, 95% CI: 1.03–1.37, *P* for trend = 0.026) (38). Another study reported that general population older than 60 years in the fourth quartile of SIRI versus the first quartile had a 1.66-fold (95% CI: 1.48–1.86) higher risk of all-cause mortality (39). Also, SIRI was found to be an independent predictor of in-hospital death in older patients with acute myocardial infarction (odds ratio [OR] = 3.948, 95% CI: 1.910–8.158) (23). In addition, this positive association between SIRI and mortality has been observed in other diseases (e.g., cancer, coronary artery disease, stroke, asthma, obesity) (20–22, 25–28).

However, we found that there were no significant differences in the risks of death between second and third tertile. Similar results in the full-adjustment models were found in older patients with heart failure for 90-day mortality (second tertile: HR = 1.31; third tertile: HR = 1.41), and 1-year mortality (second tertile: HR = 1.10; third tertile: HR = 1.19) (38). When patients were divided into four groups according to SIRI quartiles, the association in the second or third quartile lost the statistical significance in other studies (24, 27, 28, 39). We then investigated the dose–response relationship using RCS analysis, and the result showed a positive non-linear relationship between SIRI and mortality. This non-linear relationship has also been noted in patients with myocardial infarction (24), asthma (27), and heart failure (38). Furthermore, the inflection points of ln (SIRI) among these studies were 2.9 (SIRI = 18.17) and 4.6 (SIRI = 99.48) for myocardial infarction (24), and 0.67 (SIRI = 1.95) for asthma (27). In our study, the inflection point was 5.0, which was within the range reported above.

Although the underlying mechanism is unclear, several pathways could be possible explanations for the association. Firstly, our recent study indicated that hospitalized patients with high SIRI had an increased risk of DVT, which is a relatively common cause of death worldwide (40). Meanwhile, high SIRI level was also found to be an independent risk factor for pneumonia (41, 42), which was proved to be the most significant mediator contributing to the largest proportions of excess mortality following hip fracture (43). In addition, Lu et al. reported that SIRI was positively correlated with the occurrence of postoperative delirium in older patients with hip arthroplasty surgery (44). Numerous studies have confirmed that postoperative delirium in hip fracture patients was obviously associated with poor outcomes, including higher mortality (45, 46). Thus, the positive association between SIRI and mortality may be partially explained by its association with complications after hip fracture. Secondly, a recent review, which focused on the immune changes in older patients with hip fracture, concluded that hip fractures trigger a complex set of immune responses (including proinflammatory and immunosuppressive effects, also known as immune senescence or immune dysregulation), and then directly lead to patient death (47). This may be another possible reason for the association. Thirdly, since chronic, low-grade, systemic inflammation emerges with increasing age, inflammaging may also be involved in the association between SIRI and mortality. Supporting this notion, Lackner et al. found that age and the age-associated phenomenon of inflammaging seemed to be an independent risk factor aggravating and accelerating cardiac alterations following hip fracture (48).

This study has several strengths. First, all hospitalized patients were consecutively screened for inclusion, thus reducing the risk of selection bias. Second, combining automated data collection and manual review improved the quality of the data. Third, patients in our database were followed up over a long time period (a median period of 43.85 months with the longest period of 124 months), and the rate of lost to follow-up was low (5.8%), thereby we were able to assess death accurately. Lastly, a series of statistical analyses (e.g., multivariate, RCS and sensitivity analyses) were performed and obtained similar results, indicating that our findings were stable and reliable.

However, this study has certain limitations. First, although the data came from a well-maintained database, the bias of a retrospective study was inevitable. Meanwhile, the sample size was relatively small, which limited the statistical power. Also, this was a single-center study, which limited the generalizability of our findings. Therefore, further prospective studies with large sample sizes in other populations are required. Second, despite inclusion of all consecutive eligible patients with hip fracture over a 10-year study period, we could not design a matched control group because of the absence of cases without hip fracture in our database. For this reason, a comparison of our findings with non-fracture patients was not available. Third, due to the retrospective nature of the study design, some important variables influencing SIRI levels and death events could not be accessed, such as frailty status, pre-fracture functional status, social factors, the use of anti-inflammatory drugs, the development of complications, and postoperative weight-bearing status. Fourth, we were not able to routinely measure height and weight due to the inability to ambulation after hip fracture, and some patients could not remember their own height or weight, thereby resulting in missing data on BMI. Fortunately, excluding patients with missing data did not substantively change the results. Lastly, the level of SIRI fluctuates constantly, and its dynamic status has been found to be closely related with the survival of gastric cancer patients (49). In this study, the SIRI levels in most cases were measured only once at admission, which may underestimate the association because of this variability over time.

## 5 Conclusion

In conclusion, high SIRI level at admission was significantly and positively associated with high SIRI level at admission was significantly and positively associated with an increased risk of death, suggesting that SIRI may be an independent predictor of mortality in older patients with hip fracture. As a simple and practical biomarker in clinical settings, SIRI may help clinicians to identify hip fracture patients at high risk of death, which is useful to optimize treatment strategies and improve prognosis for these high-risk patients.

## Data availability statement

The raw data supporting the conclusions of this article will be made available by the authors, without undue reservation.

## Ethics statement

The studies involving humans were approved by the study protocol was approved by our Institutional Ethics Committee (Ethical approve number: 2022-04-040-K01). The studies were conducted in accordance with the local legislation and institutional requirements. The participants provided their written informed consent to participate in this study.

## Author contributions

ZF: Writing – original draft, Project administration, Methodology, Investigation, Data curation. BG: Writing – original draft, Project administration, Methodology, Investigation, Data curation. ZW: Writing – review & editing, Visualization, Software, Resources, Funding acquisition, Formal analysis, Conceptualization. XC: Writing – review & editing, Visualization, Software, Resources, Funding acquisition, Formal analysis, Conceptualization. ML: Writing – review & editing, Supervision, Software, Project administration, Formal analysis.
