# Supplementary material for: Association of systemic inflammation response index with mortality risk in older patients with hip fracture: a 10-year retrospective cohort study
Source: Front Med (Lausanne). 2024 May 22;11:1401443. doi: 10.3389/fmed.2024.1401443 (PMC11150681; doi:10.3389/fmed.2024.1401443)
Supplement: Supplementary file 1 [file Data_Sheet_1.docx]

**Supplementary Table 1.** Univariate Cox regression analyses of factors associated with mortality.

| **Variables** |  | **HR (95% *CI*)** | ***P* value** |
| --- | --- | --- | --- |
| Age (≥ 80 *vs*. < 80 years) |  | 3.591 (2.838-4.545) | <0.001 |
| Sex (male *vs*. female) |  | 1.315 (1.056-1.637) | 0.015 |
| Body mass index (*vs*. normal weight) |  |  |  |
| underweight |  | 1.684 (1.301-2.179) | <0.001 |
| overweight and obese |  | 0.712 (0.533-0.951) | 0.022 |
| Marital status (widowed *vs*. other) |  | 1.521 (1.201-1.926) | 0.001 |
| Smoking (yes *vs*. no) |  | 1.349 (1.055-1.726) | 0.017 |
| Year of admission (2020-2022 *vs*. 2013-2019) |  | 1.806 (1.367-2.385) | <0.001 |
| Length of stay (per day increase) |  | 1.014 (1.006-1.021) | 0.001 |
| Hypertension (yes *vs*. no) |  | 1.099 (0.882-1.368) | 0.401 |
| Diabetes mellitus (yes vs no) |  | 1.652 (1.291-2.114) | <0.001 |
| Coronary heart disease (yes *vs*. no) |  | 1.509 (1.024-2.223) | 0.038 |
| Atrial fibrillation (yes *vs*. no) |  | 1.972 (1.108-3.512) | 0.021 |
| Heart failure (yes *vs*. no) |  | 1.461 (0.753-2.837) | 0.262 |
| Peripheral vascular disease (yes *vs*. no) |  | 1.214 (0.452-3.257) | 0.700 |
| Cerebrovascular disease (yes *vs*. no) |  | 1.642 (1.177-2.290) | 0.004 |
| Alzheimer disease (yes *vs*. no) |  | 2.674 (1.615-4.428) | 0.001 |
| Chronic pulmonary disease (yes *vs*. no) |  | 2.138 (1.660-2.755) | <0.001 |
| Connective tissue disease (yes *vs*. no) |  | 1.368 (0.678-2.760) | 0.381 |
| Liver disease (yes *vs*. no) |  | 1.094 (0.351-3.414) | 0.877 |
| Hemiplegia (yes *vs*. no) |  | 2.045 (0.509-8.214) | 0.313 |
| Chronic kidney disease (yes *vs*. no) |  | 2.865 (1.872-4.386) | <0.001 |
| Cancer (yes *vs*. no) |  | 2.660 (1.650-4.289) | <0.001 |
| Fracture type (intertrochanteric *vs*. neck) |  | 1.599 (1.288-1.984) | <0.001 |
| Time to surgery (per day increase) |  | 1.040 (1.019-1.059) | <0.001 |
| Surgical procedure (*vs*. total hip arthroplasty) |  |  |  |
| hemiarthroplasty hip arthroplasty |  | 4.361 (2.716-7.004) | <0.001 |
| internal fixation |  | 3.523 (2.223-5.582) | <0.001 |
| Anesthetic type (general *vs*. regional) |  | 1.514 (1.200-1.910) | 0.001 |

Abbreviations: HR, hazard ratio; CI, confidence interval.

**Supplementary Table 2.** Multicollinearity analysis with variance inflation factors for multivariate Cox analyses.

| **Variables** |  | **SIRI as continuous variable** | | | **SIRI as categorical variable** | | |
| --- | --- | --- | --- | --- | --- | --- | --- |
|  |  | **Model 1** | **Model 2** | **Model 3** | **Model 1** | **Model 2** | **Model 3** |
| SIRI (continuous) |  | 1.045 | 1.054 | 1.073 |  |  |  |
| SIRI (first tertile) |  |  |  |  | 1.411 | 1.421 | 1.461 |
| SIRI (second tertile) |  |  |  |  | 1.347 | 1.357 | 1.360 |
| Age |  | 1.142 | 1.181 | 1.372 | 1.151 | 1.192 | 1.451 |
| Sex |  | 1.405 | 1.423 | 1.435 | 1.413 | 1.431 | 1.443 |
| BMI (underweight) |  | 1.435 | 1.463 | 1.474 | 1.432 | 1.462 | 1.474 |
| BMI (normal weight) |  | 1.397 | 1.402 | 1.406 | 1.399 | 1.403 | 1.410 |
| Marital status |  | 1.149 | 1.157 | 1.163 | 1.149 | 1.157 | 1.165 |
| Smoking |  | 1.357 | 1.379 | 1.387 | 1.358 | 1.380 | 1.389 |
| Year of admission |  | 1.071 | 1.136 | 1.144 | 1.082 | 1.143 | 1.155 |
| Length of stay |  | 1.059 | 1.101 | 1.599 | 1.056 | 1.096 | 1.600 |
| Diabetes mellitus |  |  | 1.134 | 1.140 |  | 1.136 | 1.142 |
| Coronary heart disease |  |  | 1.043 | 1.052 |  | 1.044 | 1.054 |
| Atrial fibrillation |  |  | 1.037 | 1.044 |  | 1.037 | 1.044 |
| Cerebrovascular disease |  |  | 1.044 | 1.054 |  | 1.046 | 1.060 |
| Alzheimer disease |  |  | 1.024 | 1.039 |  | 1.024 | 1.040 |
| Chronic pulmonary disease |  |  | 1.098 | 1.105 |  | 1.099 | 1.113 |
| Chronic kidney disease |  |  | 1.057 | 1.064 |  | 1.059 | 1.067 |
| Cancer |  |  | 1.025 | 1.029 |  | 1.026 | 1.031 |
| Type of hip fracture |  |  |  | 1.580 |  |  | 3.327 |
| Time to surgery |  |  |  | 1.724 |  |  | 1.571 |
| Surgical procedure |  |  |  | 1.454 |  |  | 1.948 |
| Anesthetic type |  |  |  | 1.042 |  |  | 1.040 |

Abbreviations: SIRI, systemic inflammation response index; BMI, body mass index; CCI, Charlson comorbidity index.

Model 1: adjusted for demographics (age, sex, body mass index, marital status, smoking, year of admission, length of stay).

Model 2: adjusted for demographics, and comorbidities (diabetes mellitus, coronary heart disease, atrial fibrillation, cerebrovascular disease, Alzheimer disease, chronic pulmonary disease, chronic kidney disease, cancer).

Model 3: adjusted for demographics, comorbidities, type of hip fracture, and surgical data (time to surgery, surgical procedure, anesthetic type).
